# Shared Decision‐Making Tool for Treatment of Perinatal Opioid Use Disorder

**DOI:** 10.1176/appi.prcp.20180004

**Published:** 2019-01-08

**Authors:** Constance Guille, Hendree E. Jones, Alfred Abuhamad, Kathleen T. Brady

**Affiliations:** ^1^ Departments of Psychiatry and Behavioral Sciences; ^2^ Departments of Obstetrics and Gynecology Medical University of South Carolina Charleston; ^3^ UNC Horizons Department of Obstetrics and Gynecology University of North Carolina at Chapel Hill Department of Psychiatry and Behavioral Sciences School of Medicine Johns Hopkins University Baltimore; ^4^ Department of Obstetrics and Gynecology Eastern Virginia Medical School Norfolk; ^5^ Ralph H. Johnson Veterans Administration Medical Center Charleston S.C.

**Keywords:** Opioid Use Disorder, Pregnancy, Buprenorphine, Methadone, Opioid Taper, Shared Decision Making

## Abstract

**Objective:**

This study examined the proportion of pregnant women with opioid use disorder (OUD) who made a decision to continue or taper pharmacotherapy for the treatment of OUD after using a shared decision‐making aid and determined whether the aid reflected the principles of the International Patient Decision Aid Standards (IPDAS).

**Methods:**

A shared decision‐making aid was developed with the IPDAS instrument to assist pregnant women with OUD receiving care at an outpatient obstetrics clinic in their decision to continue or taper pharmacotherapy for the treatment of OUD. After using the aid, the women (N=22) completed an anonymous survey about the extent to which the aid adhered to IPDAS principles.

**Results:**

Eighty‐two percent (22 of 27) of eligible participants agreed to take part in the study. After the shared decision‐making process, 95% (21 of 22) reported choosing to either continue (64%, 14 of 22) or taper (36%, 8 of 22) pharmacotherapy for the treatment of OUD. Participants agreed that they were provided with sufficient information (96%, 21 of 22), outcome probabilities (91%, 20 of 22), and decisional guidance (86%–95% [19 and 21 of 22, respectively]) to make an informed decision. Further, women reported that their choice reflected their values and preferences (77%–91% [17 and 20 of 22, respectively]).

**Conclusions:**

This shared decision aid provides a tool for providers to use with pregnant women with OUD to ensure that patients make informed treatment decisions that reflect their individual preferences and values.

**Publisher's Note:** This article is part of a special issue titled “The Opioid Crisis: Filling in the Picture.”

Shared decision making is a process in which health care providers engage patients in evidence‐based clinical decisions that support patient preferences (1). Shared decision making is widely accepted as an ideal in patient‐centered care, in which decisions are driven by well‐informed patients and reflect individual values (2). Decision aids are an effective adjunct to discussions about treatment decisions between patients and providers (3). Such aids provide explicit information about treatment options and associated harms and benefits, help clarify patient values, and provide a structured means to help patients deliberate available treatment choices. According to a Cochrane review (3) of 105 studies (31,043 participants) published through 2015, the use of patient decision aids for a range of preference‐sensitive decisions leads to increased knowledge, more accurate perception of risk, a greater number of decisions consistent with patients' values, reduced internal decisional conflict, and fewer patients remaining passive or undecided.

Shared decision‐making and patient decision aids have been used in many clinical settings and have been shown to be beneficial adjuncts to clinical care (3). Decision aids in obstetrics care have been used to help guide treatment decisions related to prenatal testing, vaginal birth after cesarean section, external cephalic version, and the use of analgesia during labor (4). Decision aids used in obstetric settings have been associated with positive effects, including reduced anxiety and decisional conflict, improved knowledge and satisfaction, and increased perception of having made an informed choice (4). Given the increased prevalence of opioid use disorder (OUD) in pregnant women (5, 6, 7, 8) in the United States, and the opportunity for treatment of the disease in conjunction with prenatal care, use of decision aids may help facilitate patient‐provider discussions of treatment options and may help ensure that patients make informed treatment choices that support their own preferences and values.

The American College of Obstetricians and Gynecologists and the American Society of Addiction Medicine recommend buprenorphine or methadone for the treatment of OUD during pregnancy. These recommendations are largely based on data that demonstrate a high risk of relapse to opioid use after tapering of methadone and buprenorphine treatment during pregnancy (9, 10, 11). Unfortunately, many women are unable to access these treatments because of the lack of availability of this type of care in their area, difficulties with transportation or child care, or concerns about child welfare consequences associated with substance use in pregnancy (12, 13, 14). Further, some women prefer not to take these medications because of risks to the newborn that are associated with use of these medications during pregnancy, such as neonatal opioid withdrawal syndrome (NOWS) (12, 13, 14).

Increasingly, obstetricians are in support of pregnant women tapering their methadone or buprenorphine, because of the low risk for poor obstetrics outcomes with a gradual taper and theoretical decreased risk for NOWS (15). However, in the largest study to date of pregnant women who tapered their buprenorphine, rates of NOWS ranged from 17.4% to 70.1%, suggesting that the risk of NOWS is not mitigated by discontinuation of buprenorphine (13). Further, the rates of relapse to illicit drug use with discontinuation of buprenorphine in that study ranged from 14.3% to 74%, with higher rates of relapse associated with higher rates of NOWS and less follow‐up care following buprenorphine taper (15). Similarly, rates of relapse to illicit drug use with discontinuation of methadone were shown to range from 41% to 96% (16), and relapse has been associated with several other poor maternal, obstetric, and newborn outcomes (8). It is, therefore, important that women be provided with balanced information about the maternal, fetal, and newborn risks of continuing or tapering methadone or buprenorphine so that they can make informed treatment decisions that are optimal for themselves and their families.

The purpose of this study was to gather feedback from pregnant women with OUD who engaged in shared decision making with their health care provider, using a decision aid designed to help facilitate an informed decision to continue or taper methadone or buprenorphine therapy during pregnancy. Specifically, the objective of this study was to determine the proportion of women who arrived at a treatment decision by following shared decision making and felt that the decision aid supported the basic principles and framework of shared decision making according to the International Patient Decision Aid Standards.

## Methods

### Materials

We developed a shared decision‐making aid using the IPDAS Instrument (IPDASi), which describes six main domains and provides a checklist of items based on a dimensional framework to guide the development of decision aids (17). According to the IPDASi, a decision aid (18) should cover six domains—supplying information about options in sufficient detail for making a specific decision, providing outcome probabilities, allowing for clarification and expression of patient values, offering structured guidance for deliberation and communication, providing evidence to support statements, and disclosing author credentials and qualifications. We used the IPDASi as a guide to construct a decision aid to assist pregnant women with OUD in choosing to continue or taper methadone or buprenorphine during pregnancy (the shared decision aid is provided in the online supplement).

Content developed for the decision aid directly corresponded to each dimension described in the IPDASi (information, probabilities, values, decision guidance, evidence, and disclosure). The decision aid was organized to facilitate conversation with patients about the decision to taper or continue methadone or buprenorphine during pregnancy. Specifically, the decision aid summarizes the treatment choices; the current literature describing what is known and not known about the maternal, fetal, and newborn risks and benefits of methadone or buprenorphine; and the risks and benefits associated with tapering methadone or buprenorphine treatment during pregnancy. Written at the eighth‐grade reading level, the decision aid is then personalized by the patient and provider to include information about the individual's risk for relapse to illicit drug use and her preferences for treatment. On a scale of 1–10, participants indicate the likelihood of their relapse to illicit drug use if they taper their buprenorphine or methadone treatment, with 10 representing the greatest likelihood for relapse. Participants are then asked to list the reasons their rating is not higher (e.g., reasons that support a decreased risk for relapse) or lower (e.g., reasons that support an increased risk for relapse). Similarly, participants are asked to list the reasons they prefer or do not prefer to take methadone or buprenorphine during pregnancy. Together, the patient and provider discuss the pros and cons of continuing or discontinuing methadone or buprenorphine treatment. The decision aid also includes suggestions related to delivery and postpartum care for pregnant women with OUD.

### Participants and Procedure

Potential participants were pregnant women with OUD taking methadone or buprenorphine who came for perinatal care from August 2015 to March 2016 at an outpatient obstetric clinic in which psychiatric services are integrated in clinical practice. Women who expressed uncertainty about continuing or tapering methadone or buprenorphine treatment during pregnancy and had been stable in recovery (defined as the absence of cravings for opioids or other drugs except for nicotine) for the prior four months were invited to participate in this study. Women reporting intravenous opioid use during the prior six months were excluded from the study. Using the decision aid, women engaged in shared decision making with their provider about whether to continue or taper buprenorphine or methadone treatment during pregnancy. Participants were then asked to complete an anonymous survey about the shared decision aid. Using a 5‐point Likert scale (i.e., strongly disagree, disagree, neutral, agree, strongly agree), participants rated their agreement with statements about the decision aid, including statements related to information presented, outcome probabilities, personal values, decisional guidance, and decisional conflict. Lastly, participants were asked if the developers of the decision aid were disclosed. A $20 gift card to Amazon.com was provided for survey participation. The Institutional Review Board of the Medical University of South Carolina approved the study, and the participants provided informed consent.

Participants received medication management and relapse prevention therapy within the outpatient obstetrics practice where they were also receiving prenatal care. After deciding to continue or taper methadone or buprenorphine, the women were seen in the obstetrics practice for therapy and medication management weekly for four weeks, every two weeks for the next four weeks, and monthly thereafter, as appropriate. Women receiving methadone continued their usual care through their methadone treatment center; methadone dose adjustments were coordinated with the provider at the methadone treatment center. Throughout treatment, self‐reported cravings for opioids or other substances, substance use via self‐report, prescription drug use, electronic health records, and urine drug screens were monitored by the prescribing provider. An increased risk of relapse (i.e., self‐reported increase in cravings) or relapse to substance use would result in the recommendation to return to a previously effective dose or to an increased dose of methadone or buprenorphine.

## Results

Among women who were eligible and approached to take part in this study, 82% (22 of 27) agreed to participate. Participants were a mean±SD of 30.1±3.28 years; 86% (19 of 22) were Caucasian. Seventy‐seven percent (17 of 22) reported being in a relationship. Of those in a relationship, 32% (7 of 22) were married, 18% (4 of 22) were engaged, 14% (3 of 22) were living with a partner, and 14% (3 of 22) were part of a couple but were not living together. Eighty‐two percent (18 of 22) reported having an annual income less than $25,000. Sixty‐four percent (14 of 22) received insurance coverage through Medicaid; 36% (8 of 22) had commercial insurance. The reading level of participants was unknown, but 68% (15 of 22) had completed high school. On average, the participants came to the integrated perinatal and psychiatry clinic at 19±2.98 weeks gestation. The median number of pregnancies among participants was three (range 1–9), and the median number of living children was one (range 0–7). On average, the participants reported having had OUD for 6.2±3.20 years; 14% (3 of 22) were being treated with methadone, and 86% (19 of 22) were being treated with buprenorphine.

Following the shared decision‐making process, 95% (21 of 22) of the women reported making a clear choice to either continue (64%, 14 of 22) or taper (36%, 8 of 22) buprenorphine or methadone. Participants agreed or strongly agreed that they were provided with sufficient medical information (96%, 21 of 22) and understood the medical information about their treatment choices (91%, 20 of 22). A majority (91%, 20 of 22) of participants agreed or strongly agreed that they were provided with evidence and risk probabilities associated with their treatment options. A majority also agreed or strongly agreed that they received decisional guidance (86%–95%, 19 and 21 of 22, respectively) and that their chosen treatment was in line with their values (77%–91%, 17 and 20 of 22, respectively) (Table 1).

**Table 1:** Women's satisfaction with a shared decision‐making tool for use in perinatal opioid use disorder^a^

Survey question	IPDASi domain	Agree or strongly agree	
		N	%
I was provided with enough information to make an informed treatment choice.	Information	20	91
I understood the medical information provided about my treatment choices.	Information	21	96
My provider discussed and provided medical information to support statements about my treatment choices.	Evidence	20	91
I understood the risk to me, my pregnancy, and newborn with taking medication such as buprenorphine or methadone during pregnancy.	Evidence and probability	20	91
I understood the risk to me, my pregnancy, and newborn with not taking medication such as buprenorphine or methadone during pregnancy.	Evidence and probability	20	91
My treatment choice supported my values.	Values	17	77
My treatment choice supported my preferences.	Values	20	91
I was able to discuss which treatment choice was right for me.	Values	19	86
I was able to weight the pros [positives] and cons [negatives] of treatment choices.	Decision guidance	20	91
I felt that the treatment choices were made in partnership with my provider.	Decision guidance	19	86
I made a decision to either continue or discontinue my methadone or buprenorphine.	Decisional conflict	21	95
The developers of the decision aid were clear and disclosed during the decision‐making process.	Disclosure	21	95

^a^ IPDASi, International Patient Decision Aid Standards Instrument. Survey responses were anonymous.

Among women taking methadone, 33% (1 of 3) decided to taper their methadone. Among women taking buprenorphine, 37% (7 of 19) decided to taper their buprenorphine. The most common reasons for tapering methadone or buprenorphine were concern about NOWS (100%, 8 of 8), potential involvement of the Department of Social Services (75%, 6 of 8), stigma (75%, 6 of 8), and having to withdraw during the postpartum period because of their inability to afford the medication postpartum (63%, 5 of 8). The most common reasons for continuing methadone or buprenorphine treatment were increased risk for relapse to illicit drug use (79%, 11 of 14), belief that opioid withdrawal during pregnancy would harm the baby (65%, 9 of 14), and concern about worsening mood and/or pain (43%, 6 of 14).

## Discussion

A shared decision‐making tool was created in accordance with the IPDAS to assist women in recovery from OUD with their decision to continue or taper buprenorphine or methadone treatment during pregnancy. Following use of the decision aid, 95% of the participants, who were initially ambivalent about their treatment choice, made a decision to continue or taper buprenorphine or methadone. Most of the women agreed that the decision aid supported the basic principles and intent of shared decision making and provided enough information to make an informed treatment choice that supported their values and preferences.

Our findings suggest that this shared decision tool has the potential to improve care of pregnant women with OUD and is consistent with the positive benefits of other shared decision aids used in obstetrics (4) and other clinical specialties (3). The benefits of such aids shown in prior studies and in our study include reduced decisional conflict, improved knowledge, and increased perception of having made an informed decision in partnership with a health care provider. These findings are important given the increasing prevalence of OUD in pregnancy (8); the complexity of the maternal, obstetric, and newborn risks associated with each treatment choice; and the lack of definitive data to guide treatment decisions (19). Providing balanced information about the maternal, fetal, and newborn risks associated with untreated maternal OUD and the risks associated with its treatments is paramount to ensuring that women can make optimal treatment choices for themselves and their families.

As expected, the most common reason the participants chose to continue methadone or buprenorphine was the perceived risk of relapse to illicit drug use. Knowing this risk and the potential harms associated with relapse can help mothers feel less conflicted about their use of methadone or buprenorphine treatment and the associated risks, including NOWS. Knowing that they made the best choice possible can be helpful to mothers coping with a newborn with NOWS. Interestingly, 65% of the women believed that opioid taper during pregnancy was potentially harmful. This belief may reflect a historical practice of cautioning against opioid taper during pregnancy because of an increased risk for poor obstetric outcomes (20). Current practice recognizes that the poor obstetric outcomes in earlier studies likely were attributable to relapse to illicit drug use, as opposed to the effect of tapering methadone or buprenorphine (16, 21, 22, 23). The use of shared decision making allowed for clarification of misinformation regarding this older practice. Forty‐three percent of the women were concerned that discontinuing buprenorphine or methadone might worsen their mood or pain symptoms. This finding highlights the importance of addressing common comorbid conditions in women with OUD.

As anticipated, the most common reason women chose to taper methadone or buprenorphine was the perceived risk of NOWS. Although risk of NOWS is an important consideration, the advantage of the shared decision‐making tool is that the decision to taper methadone or buprenorphine was not driven solely by this risk, and NOWS was considered as one variable in the overall treatment decision. Seventy‐five percent of women who chose to discontinue methadone or buprenorphine expressed concern about the potential involvement of the Department of Social Services as well as how they would be viewed by medical staff during delivery if they were taking methadone or buprenorphine. These perceptions are concerning because they may deter women from receiving the standard of care for OUD. Sixty‐three percent of women planned to taper their buprenorphine or methadone during pregnancy because of their inability to afford the medication after delivery and because they felt that the risks associated with withdrawal and the potential for relapse would be greater postpartum than during pregnancy. Although methadone is often funded by Opioid State Targeted Response grants and buprenorphine is covered by most insurance, including Medicaid, during pregnancy, many women are not afforded these same benefits postpartum. This finding highlights an important treatment gap and suggests that women may be discontinuing methadone or buprenorphine because of the inability to access or pay for these lifesaving treatments. These findings are concerning given the rising mortality rates from opioid overdose in the United States (24) as well as rising rates of maternal mortality (25).

While participants overall provided positive feedback on the shared decision‐making tool, caution should be taken in interpreting the potential benefit of the tool because of this study's lack of randomization. It is possible that patients would have equally benefited from discussing treatment choices with their providers without the decision tool. However, the use of a decision aid helps ensure that information is not inadvertently omitted, and patient information sheets have been shown to improve patient medical knowledge (26). Additionally, use of a validated rating scale to assess the utility or benefit of the decision aid would have strengthened the feedback gathered in this study. To our knowledge, no validated scale to assess fidelity to the IPDAS framework is available. The study could have benefited also from the addition of a validated anxiety measure or patient satisfaction survey, because prior studies conducted with pregnant women have demonstrated reduced anxiety and increased patient satisfaction with the use of shared decision aids (4). Given that the women perceived they were able to make an informed decision after the shared decision‐making process, however, provides some support that the decision aid achieved its intent.

The study participants represent those who were able to access prenatal care and substance use disorder treatment within our outpatient obstetrics practice. Future studies are needed to investigate how access to care and availability of care for pregnant and postpartum women with OUD influence treatment decisions and retention in treatment.

## Conclusions

Given the dramatic increase in opioid use during pregnancy in the United States, this shared decision aid provides a timely clinical tool for providers to use with pregnant women with OUD. This study demonstrates the utility of this shared decision‐making aid in reducing decisional conflict about continuing or tapering buprenorphine or methadone treatment during pregnancy and ensuring that women with OUD have the opportunity to make informed, evidence‐based decisions that support their individual values and preferences and that are ultimately optimal for themselves and their families.

## Supporting information

Supplementary Material
