# Supplementary material for: Shared Decision‐Making Tool for Treatment of Perinatal Opioid Use Disorder
Source: Psychiatr Res Clin Pract. 2019 Jan 8;1(1):27–31. doi: 10.1176/appi.prcp.20180004 (PMC9176026; doi:10.1176/appi.prcp.20180004)
Supplement: Supplementary file 1 — Supplementary Material [file RCP2-1-27-s001.pdf]

A Shared Decision-Making Tool for the
Treatment of Perinatal Opioid Use Disorder (OUD)

Who is this treatment decision aid for?

This decision aid is for pregnant women who are stable in recovery from their Opioid Use Disorder (OUD). The following information is to help pregnant women decide which medication approach is best for the treatment of OUD. Specifically, the following is to help women decide to either continue or taper Buprenorphine or Methadone during pregnancy.

What are the current treatment recommendations for pregnant women with OUD?

Several professional organizations(1-6) recommend Methadone or Buprenorphine as part of a comprehensive treatment plan for pregnant women with OUD. This recommendation is based on data that demonstrate that pregnant women who taper Methadone or Buprenorphine are at high risk for relapse to drug use and that drug use causes more harm to women, their pregnancy, fetus and newborn compared to treatment with Methadone or Buprenorphine.(1,2)

What do I need to know in order to decide if taking Methadone or Buprenorphine is right for me?

It is important that you understand the risks of taking Methadone or Buprenorphine during pregnancy. It is equally as important that you understand the risk of relapse to drug use if you taper Methadone or Buprenorphine as well as the potential harms associated with drug use during pregnancy.

We encourage you to read the following and talk with your provider about the risks of Methadone and Buprenorphine and the risks of not taking these medications, in general, and for you as an individual, so that you can make an informed decision about the treatment of OUD that is best for you and your family.

Risks of Methadone or Buprenorphine:

Taking Buprenorphine or Methadone during pregnancy has been associated with: 1) Prematurity; 2) Low birth weight; and 3) Newborn Opioid Withdrawal Syndrome (NOWS).

Prematurity

Prematurity is defined as birth prior to 37 weeks gestation. The rate of preterm birth in the United States is 9.63%.(7) Preterm birth is associated with newborn death, breathing and feeding difficulties as well as cerebral palsy, developmental delays, vision problems and hearing problems.(7) Estimates vary, but approximately 7-19% of women taking Buprenorphine or Methadone during pregnancy have experienced preterm delivery.(8) You can decrease your risk of preterm birth by quitting smoking.

Low Birth Weight

Low birth weight is birth weight less than 2.5kg (2500g). The rate of low birth weight in the United States is 9.47%.(7) Low birth weight is associated with adult chronic medical conditions, such as diabetes, hypertension and heart disease.(9) Estimates vary, but approximately 2.1-9.3% of women taking Buprenorphine or Methadone during pregnancy will have newborns with low birth weight.(10) You can decrease your risk of low birth weight by quitting smoking.

Newborn Opioid Withdrawal Syndrome (NOWS)

Both Methadone and Buprenorphine are associated with Newborn Opioid Withdrawal Syndrome (NOWS) described below. Approximately 60% of newborns exposed to Methadone or Buprenorphine during pregnancy will experience NOWS.(11,12) Symptoms of NOWS often appear within 1-3 days after birth but can take up to 1 week to appear.

The following is a list of the most common symptoms of NOWS:

- High-pitched cry
- Jitteriness
- Tremors
- Generalized convulsions
- Sweating
- Fever
- Mottling of skin
- Excessive sucking or rooting
- Poor feeding
- Vomiting
- Diarrhea

All newborns exposed to Methadone or Buprenorphine or potentially any opioid during pregnancy will be monitored for NOWS following delivery for approximately 4-7 days. Newborns with NOWS will be treated with medication and/or an opioid medication. Opioid medication will be tapered and this process can take 3-6 weeks or longer. It is therefore possible that you will be discharged from the hospital prior to your newborn, even if your newborn is just being monitored for NOWS, unless your hospital is able to make other arrangements for your stay.

You can reduce the risk and/or severity of NOWS by:

- Abstaining from drug use
- Quitting smoking
- Discontinuing or reducing the use of some medications [e.g., benzodiazepines, sedative hypnotics, selective serotonin reuptake inhibitors (SSRIs)] as appropriate during pregnancy

- Breastfeeding
- Skin-to-skin contact with your newborn

The severity of NOWS and the amount of medication and hospital stay needed to treat newborn withdrawal may be less in newborns that have been exposed to Buprenorphine compared to Methadone during pregnancy. It does not appear that reducing your dose or discontinuing your dose of Methadone or Buprenorphine eliminates your risk of NOWS. Studies where women have discontinued Buprenorphine still have newborns with NOWS.(13) The risk of NOWS in these women seems to be higher in women that relapse to drug use [70.1%] compared to women that do not relapse to drug use [17.4%].(13)

Risk of Relapse to Drug Use in Pregnancy:

The risk for relapse to drug use while on Methadone or Buprenorphine and engaged in a comprehensive addiction treatment program is approximately 9-15%.(8)

The risk for relapse to drug use when Methadone is tapered in women prior to the prescription opioid epidemic [and the primary opioid of use included heroin] is 41-96%.(14-18)

The risk for relapse to drug use when Methadone or Buprenorphine is tapered in women during the prescription opioid epidemic [and the primary drug of use was prescription opioids and/or heroin] is 17-74%.(13,19-22) The lower proportion of women that relapsed [17%] occurred in women that underwent longer tapers [e.g., 8-16 weeks] and had more intensive follow-up care [Monday- Friday intensive behavioral health program].(13) The higher proportion of women that relapsed [74%] occurred in women that underwent shorter tapers and had little follow-up care. The risk of NOWS appears to be higher in women that relapse to drug use [70.1%] compared to women that do not [17.4%].(13)

Risks of Drug use in Pregnancy:

Drug use in pregnancy has been associated with a number of poor maternal, fetal, obstetric and newborn outcomes.(23) In comparison to women without opioid abuse or dependence, women with opioid abuse or dependence were more likely to have the following complications at the time of delivery:(23)

- Death
- Cardiac arrest [heart attack]
- Intrauterine growth restriction
- Placental abruption
- Length of stay >7 days
- Preterm birth
- Oligohydramnios
- Transfusion
- Stillbirth
- Premature rupture of membranes
- Cesarean delivery
- Severe preeclampsia or eclampsia
- Anesthesia complications

Some states require that the Department of Child and Family Services be notified if a woman is using drugs during pregnancy. In some states it is illegal to use drugs during pregnancy and doing so can result in arrest or removal of child custody. It is important to know your states reporting requirements and laws related to drug use in pregnancy. A helpful resource is: https://projects.propublica.org/graphics/maternity-drug-policies-by-state

Risk of relapse to drug use for you as an individual:

Some things to consider in evaluating your risk for relapse are if you have relapsed to drug use while not taking Methadone or Buprenorphine in the past and/or in a previous pregnancy. Do you have friends and family that support your recovery? Are you able to attend and engage in addiction treatment services and/or a 12 step facilitated program?

On a scale of 1-10, with 10 being the most likely to relapse to drug use, how likely do you think you would relapse to drug use if you were not taking Methadone or Buprenorphine (circle a number below)?

1 2 3 4 5 6 7 8 9 10

Why is your rating not higher or lower?

Please list your reasons why you are likely or unlikely to relapse to drug use.

**Reasons I'm likely to not relapse**<br><br>**(Reasons why your rating is not higher)**	**Reasons I am likely to relapse**<br><br>**(Reasons why rating is not lower)**

On a scale of 1-10, with 10 being the strongest preference, rate your preference for taking Methadone or Buprenorphine during pregnancy (circle a number below).

1 2 3 4 5 6 7 8 9 10

Please list reasons why you prefer or prefer not to take Methadone or Buprenorphine.

**Do not prefer to take**<br>**Methadone or Buprenorphine**<br>**(Reasons why your rating is not higher)**	**Prefer to take**<br>**Methadone or Buprenorphine**<br>**(Reasons why rating is not lower)**

Continuing Methadone or Buprenorphine:

If you prefer to continue taking Methadone or Buprenorphine during pregnancy and/or suspect that your risk for relapse to drug use is high if you were to taper this medication, we

would recommend that you continue Methadone or Buprenorphine. We would also recommend that you work with your provider to find other ways to reduce your risk of NOWS by abstaining from drug use, quitting smoking, and/or discontinuing or minimizing the use of other medications [benzodiazepines, hypnotics, SSRIs] as appropriate. You should work with your provider to find the lowest effective dose of Methadone or Buprenorphine needed to reduce cravings, urge to use drugs and/or use of drugs. It does not appear that reducing your dose of Methadone or Buprenorphine reduces your risk of NOWS. Using a lower dose that is not effective for the treatment of your OUD incurs the risk of both the medication and the risk for relapse and risk associated with drug use.

You may also want to consider breastfeeding and skin-to-skin contact with your newborn as this may reduce the severity of NOWS. You may want to consider visiting the hospital where you will deliver to meet the staff and learn about how they will monitor and, if appropriate, treat NOWS. It will also be important to know about your hospitals drug testing procedures or potential for consultation with the Department of Child and Family Services at the time of delivery.

Discontinuing Methadone or Buprenorphine:

If you have a strong preference for discontinuing Methadone or Buprenorphine and/or suspect that your risk for relapse to drug use is low, we would recommend that you work closely with your provider(s) to taper this medication over weeks to months with frequent follow-up care. Pregnant women that discontinued their Buprenorphine had a lower risk of relapse [17%] if they received intensive behavioral health care.(13)

In an effort to reduce your risk for relapse we recommend that you taper your Buprenorphine slowly and participate in behavioral health treatment including relapse prevention

and/or a 12-step facilitated program. We recommend that if you relapse to drug use, or experience increased cravings and/or a strong desire or urge to use drugs, you should return to the prior effective dose of Buprenorphine or Methadone to reduce drug use and/or cravings. You may also want to consider breastfeeding and skin-to-skin contact with your newborn as this may reduce the severity of NOWS. You may want to consider visiting the hospital where you will deliver to meet the staff and learn about how they will monitor and, if appropriate, treat NOWS. It will also be important to know about your hospitals drug testing procedures or potential for consultation with the Department of Child and Family Services at the time of delivery.

Planning for pain management:

In the event of a cesarean section, you will likely be prescribed an opioid medication consistent with the standard of care for pain management following a cesarean section. For many people with an OUD this can be a ‘trigger’ for relapse to drug use. Asking trusted family or friends to hold and dispense this medication for you can help reduce pain associated with surgery and prevent you from overusing this medication.

Review decision aid throughout pregnancy:

This decision aid can be reviewed throughout your pregnancy and updated as new information about your treatment and recovery from OUD becomes available.

Planning for after delivery:

It is also important to plan for the postpartum period. If you are taking Methadone and Buprenorphine during pregnancy, will this medication be continued postpartum and, if so, who will prescribe this medication and how will the medication be paid for? If you are not taking Methadone or Buprenorphine during pregnancy, will you restart this medication postpartum and, if so, who will prescribe this medication and how will the medication be paid for?

Acknowledgements:

The efforts of Dr. Constance Guille (1K23DA039318-01) are funded by the National Institute on Drug Abuse. Dr. Kathleen T. Brady's effort was funded by R25 DA020537, UL1 TR00006205S1, P50 DA016511, U10 DA01372, and K12 HD055885. The funding sources had no role in the preparation or writing of this shared decision making aid.

References:

(1) ACOG Committee on Health Care for Underserved Women, American Society of Addiction Medicine: ACOG committee opinion no. 524: Opioid abuse, dependence, and addiction in pregnancy. Obstet Gynecol 2012;119(5):1070-1076

(2) ACOG Committee on Obstetric Practice, American Society of Addiction Medicine: Committee opinion no. 711 summary: Opioid use and opioid use disorder in pregnancy. Obstet Gynecol 2017;130(2):488-489

(3) Guidelines for the identification and management of substance use and substance use disorders in pregnancy. Geneva, World Health Organization (WHO). http://www.who.int/substance_abuse/publications/pregnancy_guidelines/en/. Accessed Feb 15, 2018

(4) A collaborative approach to the treatment of pregnant women with opioid use disorders. HHS Publication No (SMA) 16-4978. Rockville, MD, Substance Abuse and Mental Health Services Administration, 2016

(5) Kampman K, Jarvis M: American society of addiction medicine (ASAM) national practice guideline for the use of medications in the treatment of addiction involving opioid use. J Addict Med 2015;9(5):358-367

(6) Guidelines on drug prevention and treament for girls and women. Vienna, United Nations Office on Drugs and Crime. http://www.unodc.org/documents/drug-prevention-and-treatment/unodc_2016_drug_prevention_and_treatment_for_girls_and_women_E.pdf. Accessed Feb 15, 2018

(7) Birthweight and gestation. Atlanta, Centers for Disease Control & Prevention. https://www.cdc.gov/nchs/fastats/birthweight.htm. Accessed Feb 15, 2018

(8) Jones HE, Kaltenbach K, Heil SH, et al: Neonatal abstinence syndrome after methadone or buprenorphine exposure. New Engl J Med 2010;363(24):2320-2331
(9) Goldenberg RL, Culhane JF: Low birth weight in the united states. Am J Clin Nutr 2007;85(2):584S-590S
(10) Nørgaard M, Nielsson MS, Heide-Jørgensen U: Birth and neonatal outcomes following opioid use in pregnancy: A danish population-based study. Subst Abuse Res Treat 2015;9(Suppl 2):5-11
(11) Patrick SW, Schumacher RE, Benneyworth BD, Krans EE, McAllister JM, Davis MM: Neonatal abstinence syndrome and associated health care expenditures in the united states, 2000-2009. JAMA 2012;307(18):1934-1940
(12) Patrick SW, Dudley J, Martin PR, et al: Prescription opioid epidemic and infant outcomes. Pediatrics 2015;135(5):842-850
(13) Bell J, Towers CV, Hennessy MD, Heitzman C, Smith B, Chattin K: Detoxification from opiate drugs during pregnancy. Am J Obstet Gynecol 2016;215(3):374.e1-374.e6
(14) Blinick G, Wallach RC, Jerez E, Ackerman BD: Drug addiction in pregnancy and the neonate. Am J Obstet Gynecol 1976;125(2):135-142
(15) Maas U, Kattner E, Weingart-Jesse B, Schäfer A, Obladen M: Infrequent neonatal opiate withdrawal following maternal methadone detoxification during pregnancy. J Perinat Med 1990;18(2):111-118
(16) Dashe JS, Jackson GL, Olscher DA, Zane EH, Wendel GD: Opioid detoxification in pregnancy. Obstet Gynecol 1998;92(5):854-858
(17) Luty J, Nikolaou V, Bearn J: Is opiate detoxification unsafe in pregnancy? J Subst Abuse Treat 2003;24(4):363-367

(18) Jones HE, O'Grady KE, Malfi D, Tuten M: Methadone maintenance vs. methadone taper during pregnancy: Maternal and neonatal outcomes. Am J Addict 2008;17(5):372-386

(19) Lund IO, Fitzsimons H, Tuten M, Chisolm MS, O'Grady KE, Jones HJ: Comparing methadone and buprenorphine maintenance with methadone-assisted withdrawal for the treatment of opioid dependence during pregnancy: Maternal and neonatal outcomes. Subst Abuse Rehabil 2012;3(Suppl 1):17-25

(20) Welle-Strand GK, Skurtveit S, Tanum L, et al: Tapering from methadone or buprenorphine during pregnancy: Maternal and neonatal outcomes in norway 1996-2009. Eur Addict Res 2015;21(5):253-261

(21) Dooley R, Dooley J, Antone I, et al: Narcotic tapering in pregnancy using long-acting morphine: An 18-month prospective cohort study in northwestern ontario. Can Fam Phys 2015;61(2):e88-e95

(22) Stewart RD, Nelson DB, Adhikari EH, et al: The obstetrical and neonatal impact of maternal opioid detoxification in pregnancy. Am J Obstet Gynecol 2013;209(3):267.e1-267.e5

(23) Maeda A, Bateman BT, Clancy CR, Creanga AA, Leffert LR: Opioid abuse and dependence during pregnancy: Temporal trends and obstetrical outcomes. Anesthesiology 2014;121(6):1158-1165
